# Microstructure and Mechanical Properties of Graphene Oxide-Reinforced Titanium Matrix Composites Synthesized by Hot-Pressed Sintering

**DOI:** 10.1186/s11671-019-2951-9

**Published:** 2019-03-29

**Authors:** Jingqi Liu, Ning Hu, Xuyang Liu, Yaolu Liu, Xuewei Lv, Liangxiao Wei, Shoutao Zheng

**Affiliations:** 1 College of Aerospace Engineering, Chongqing University, Chongqing, 400044 People’s Republic of China; 2 College of Materials Science and Engineering, Chongqing University, Chongqing, 400044 People’s Republic of China

**Keywords:** Titanium matrix composites, Graphene oxide, Hot-pressed sintering, TiC

## Abstract

Ti matrix composites reinforced with 1–5 wt% graphene oxide (GO) were prepared by hot-pressed sintering in argon atmosphere. The effect of sintering temperature on the microstructures and mechanical properties of the composite was also evaluated. The results show that TiC nanoparticles were formed in situ as interfacial products via the reaction between Ti and GO during sintering. With increases in GO content and sintering temperature, the amount of TiC increased, improving the mechanical properties of the composites. GO was also partly retained with a lamellar structure after sintering. The composite reinforced with 5 wt% GO exhibited a hardness of 457 HV, 48.4% higher than that of pure Ti at 1473 K. The Ti-2.5 wt% GO composite sintered at 1473 K achieved a maximum yield stress of 1294 MPa, which was 62.7 % higher than that of pure Ti. Further increasing the GO content to 5 wt% led to a slight decrease in yield stress owing to GO agglomeration. The fracture morphology of the composite reinforced with GO exhibited a quasi-cleavage fracture, whereas that of the pure Ti matrix showed a ductile fracture. The main strengthening mechanism included grain refinement, solution strengthening, and dispersion strengthening of TiC and GO.

## Introduction

The increasing demand for lightweight and high-performance materials for the aerospace industry in recent years has led to the development of metal matrix composites (MMCs). As typical MMCs, titanium matrix composites (TMCs) have been regarded as potential candidates because of their outstanding specific strength, wear resistance, and high-temperature-performance. The refractory ceramics (TiC [1, 2], SiC [3], and TiB [4, 5]) as well as the SiC [6] fiber with a high melting point, excellent oxidative stability, and good thermal stability have generally been regarded as ideal reinforcements. However, the toughness of the matrix can be reduced owing to the inherent brittleness of the ceramic reinforcement. Fiber-reinforced TMCs are also limited by the anisotropy of the fiber, leading to an unstable performance.

Owing to their low density and excellent properties, carbonaceous nanomaterials, including carbon nanotubes and graphene, have drawn considerably more attention as reinforcement to achieve requirements such as light weight and high strength for TMCs. Graphene consisting of a single-atom layer of sp^2^-hybridized carbon atoms has a large theoretical specific surface area of 2630 m^2^/g [7]. In recent years, graphene as a reinforcement has been widely used to improve the matrix performance owing to its extraordinary electrical, thermal, and mechanical properties [8–10]. Yan et al. [11] fabricated an aluminum composite reinforced with 0.5 wt% graphene nanoflakes (GNFs) by hot isostatic pressing at 1073 K. The results indicated that the tensile strength increased from 214 MPa of pure aluminum to 319 MPa by filling 0.5 wt% GNFs. Li et al. [12] used 0.8 vol.% Ni nanoparticle-decorated graphene nanoplatelets as reinforcing components to prepare Cu matrix composites by spark plasma sintering; these composites exhibited ultimate tensile strength 43% higher than that of pure Cu. Gao et al. [13] reported that the highest ultimate tensile strength, Vickers hardness, and thermal conductivity were achieved when 0.3 wt% graphene was added into the copper matrix. However, the aforementioned properties could not be further improved even when the graphene content continued to increase. A similar phenomenon was reported by Song et al. [14], which suggested that the mechanical properties of the composite reached the maximum when 0.5 wt% multilayer graphene was added in the titanium matrix. The further improvement in the performance of the composites with additional reinforcement was limited because of the strong agglomeration of nano-carbon materials. Various attempts, including ultrasonic stirring [15], high-energy ball milling [16], and surface activation treatment [17], have been developed to improve the dispersibility of reinforcements in the matrix; however, no evident improvement has been observed.

Graphene oxide (GO) is an important derivative of graphene and contains various oxygen functional groups (hydroxyl, carboxyl acid, and epoxy) on the surface and sheet edges, thereby improving dispersibility in solvents [18–20]. Kwon et al. [21] fabricated AlMg5-1 vol.% GO composites by powder metallurgy; the ultimate tensile strength and macrohardness were about twice that of AlMg5 alloys under similar conditions. Lin et al. [22] prepared Fe matrix composites with single-layer graphene oxide by laser heating; the results indicated that the surface microhardness of Fe-2 wt% GO composites increased by 93.5% relative to that of pure iron. However, few studies have reported on the use of GO as a reinforcement to strengthen the titanium matrix. In the present study, the TMCs reinforced with GO at varying contents were prepared by hot-pressed sintering. The effect of sintering temperature on the microstructures and mechanical properties of the composite were also evaluated in detail.

## Methods/Experimental

### Synthesis of GO

GO was prepared using the modified Hummers’ method [23] with graphite as a raw material. Graphite powder was first expanded by intercalation-expansion at room temperature before oxidation [24]. The specific surface area obtained with the aforementioned approach was one order of magnitude higher than that obtained using the traditional thermally expanded method. Specifically, 1.0 g graphite powder (+ 325 mesh, purity > 99.95%, Aladdin) and an intercalant with 12.75 g CrO_3_ (Chuandong Chemical Industry, China) were added into 10.5 mL hydrochloric acid (37 wt%, Chuandong Chemical Industry, China). The mixture was stirred for 2 h at room temperature to obtain CrO_3_-based graphite intercalation compound (GIC) flakes. The GIC flakes were then washed with deionized water to remove the excess reactant, and then immersed in reactive species 60 mL H_2_O_2_ (30%, Chuandong Chemical Industry, China) for 12 h. The chemically expanded graphite (CEG) was ultimately washed with deionized water to remove the residual H_2_O_2_ and chromium salt. The chemical reactions during intercalation and expansion are described as follows:

1 $$ {\mathrm{CrO}}_3+2\mathrm{HCl}={\mathrm{CrO}}_2{\mathrm{Cl}}_2+{\mathrm{H}}_2\mathrm{O} $$

2 $$ 2{\mathrm{Cr}\mathrm{O}}_2{\mathrm{Cl}}_2+3{\mathrm{H}}_2\mathrm{O}={\mathrm{H}}_2{\mathrm{Cr}}_2{\mathrm{O}}_7+4\mathrm{HCl} $$

3 $$ {\mathrm{Cr}}_2{\mathrm{O}}_7^{2-}+2{\mathrm{H}}^{+}+4{\mathrm{H}}_2{\mathrm{O}}_2=2{\mathrm{Cr}\mathrm{O}}_5+5{\mathrm{H}}_2\mathrm{O} $$

4 $$ 2{\mathrm{Cr}\mathrm{O}}_5+6{\mathrm{H}}^{+}+7{\mathrm{H}}_2{\mathrm{O}}_2=2{\mathrm{Cr}}^{3+}+10{\mathrm{H}}_2\mathrm{O}+7{\mathrm{O}}_2\uparrow $$

The expansion was driven by the generated O_2_ gas within the interlayer of the GIC flakes. The as-prepared CEG was incorporated with a 9:1 mixture of concentrated H_2_SO_4_/H_3_PO_4_ (153.3 mL) and KMnO_4_ (6 g) in an ice water bath and then stirred for 4 h at 323 K. After the aforementioned mixture was cooled to room temperature, 200 mL ice water and 15 mL H_2_O_2_ were added to resolve insoluble impurities. Subsequently, GO was deposited after adding 30 mL hydrochloric acid. After 12 h, the concentrate solution of GO was prepared by washing the deposited GO via centrifugation until the pH of the supernatant liquid exceeded 5.

### Fabrication of the Ti/GO composite powders

Commercial pure Ti powders (Quanxing Titanium Industry, China) were used as starting materials. The chemical analysis of pure Ti is presented in Table 1. To obtain the optimal distribution of GO sheets in the Ti matrix, the as-prepared GO concentrate solution was diluted with ethyl alcohol (95%, Chuandong Chemical Industry, China). Ti powders were added into the diluted GO solution and ultrasonically dispersed for 10 min to obtain a uniform mixture. The solution was then stirred into a slurry in a semi-dried state in a water bath at 333 K to prevent the separation of Ti and GO resulting from the density difference. The slurry was completely dried in a vacuum oven at 333 K for more than 12 h, and the dried mixture was ground for 10 min to obtain a uniform composite.

**Table 1:** Chemical analysis of Ti powder

Element	Ti	H	O	N	C	Fe	Si
Content (%)	99.5	0.04	0.21	0.06	0.06	0.05	0.06

### Consolidation of Composites

**Fig. 1:**
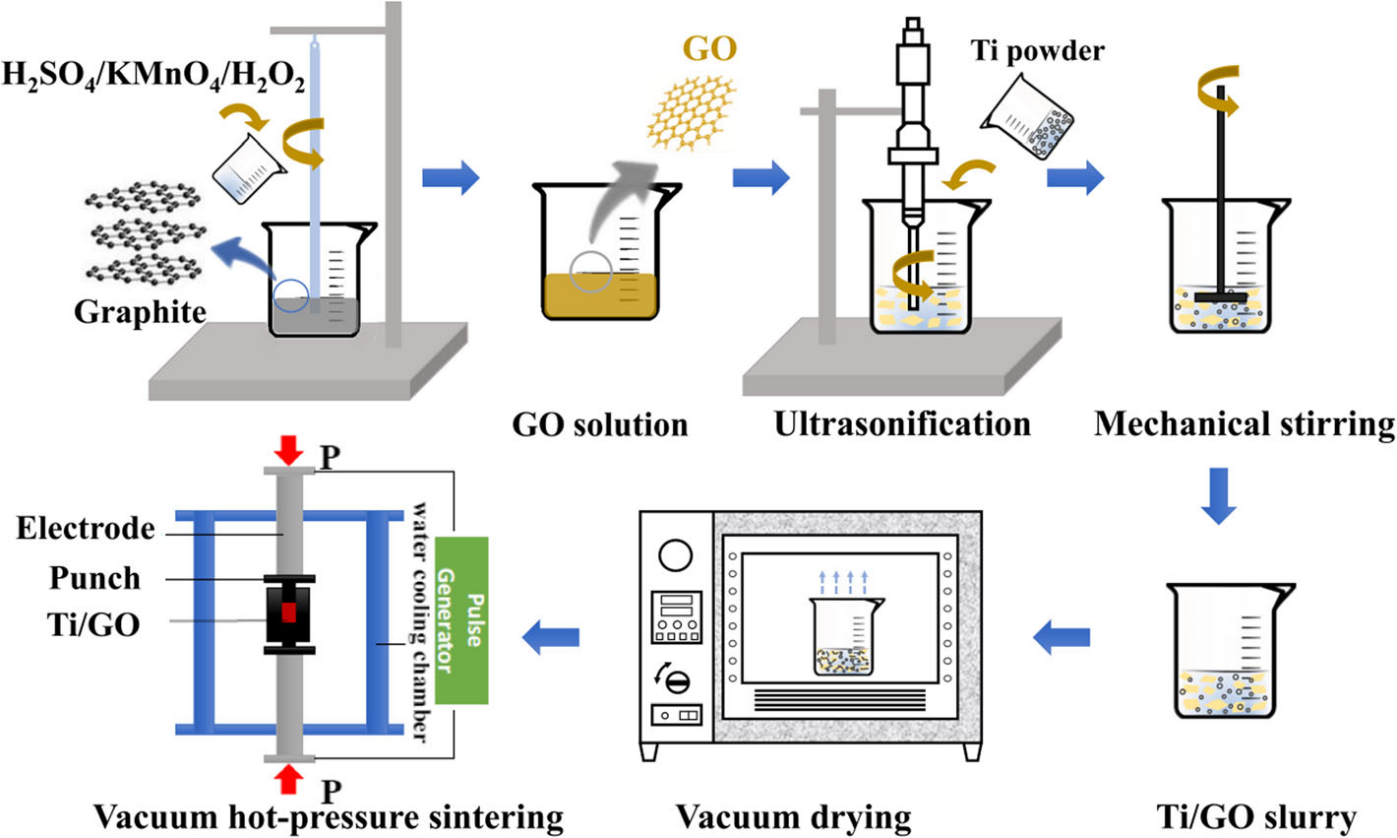
Schematic of Ti/GO composite preparation

The obtained Ti/GO mixed powders were loaded into a graphite die with an internal diameter of 15 mm and then placed in a hot-pressing furnace (JVPF-150, Shenyang Jinyan New Material Preparation Technology Co. Ltd.) with a flowing argon atmosphere. The compact was sintered at 1073 and 1473 K with a heating rate of 15 K/min for 30 min under 50 MPa pressure. The hot-pressed samples were cooled to room temperature in the furnace with a cooling rate of less than 20 K/min. The schematic of the preparation process of the Ti/GO composite is shown in Fig. 1. The sintered samples were cut into mechanical testing specimens, and the surfaces were polished to 1 μm.

### Characterization

The Chemical structure of GO was measured by Raman spectrometry (LabRAM HR Evolution, HORIBA Jobin Yvon S.A.S), X-ray photoelectron spectroscopy (XPS, ESCALAB250Xi, Thermo Fisher Scientific), and Fourier-transform infrared spectroscopy (FT-IR, Nicolet iN10, Thermo Fisher Scientific). The phase composition of the sintered samples was measured by X-ray diffraction (XRD, D2 PHASER, BRUKER). The thermal stability of GO was measured by thermogravimetric analysis (TGA, TG, 209 F3 Tarsus, NETSCH). Morphological and elemental analysis of the mixed powder and sintered composites were conducted by scanning electron microscopy (SEM), transmission electron microscopy (TEM, FEI Talos F200S G2, Thermo Fisher Scientific Inc.), and energy-dispersive spectrometry (EDS, TESCAN VEGA 3 LMH, TESCAN). The thickness of GO was measured by atomic force microscopy (AFM, Asylum Research MFP-3D-BIO, Oxford Instruments Co). The Vickers hardness, compressive strength, and thermal conductivity of the samples were measured using a microhardness tester (HX-1000TM/LCD, Shanghai Taiming Optical Instrument Co. Ltd.), a material testing machine (MTS 858, MTS), and a laser thermal conductivity testing instrument (LFA457, Netzsch, Ltd.), respectively.

## Results and Discussion

### Characterization of GO

**Fig. 2:**
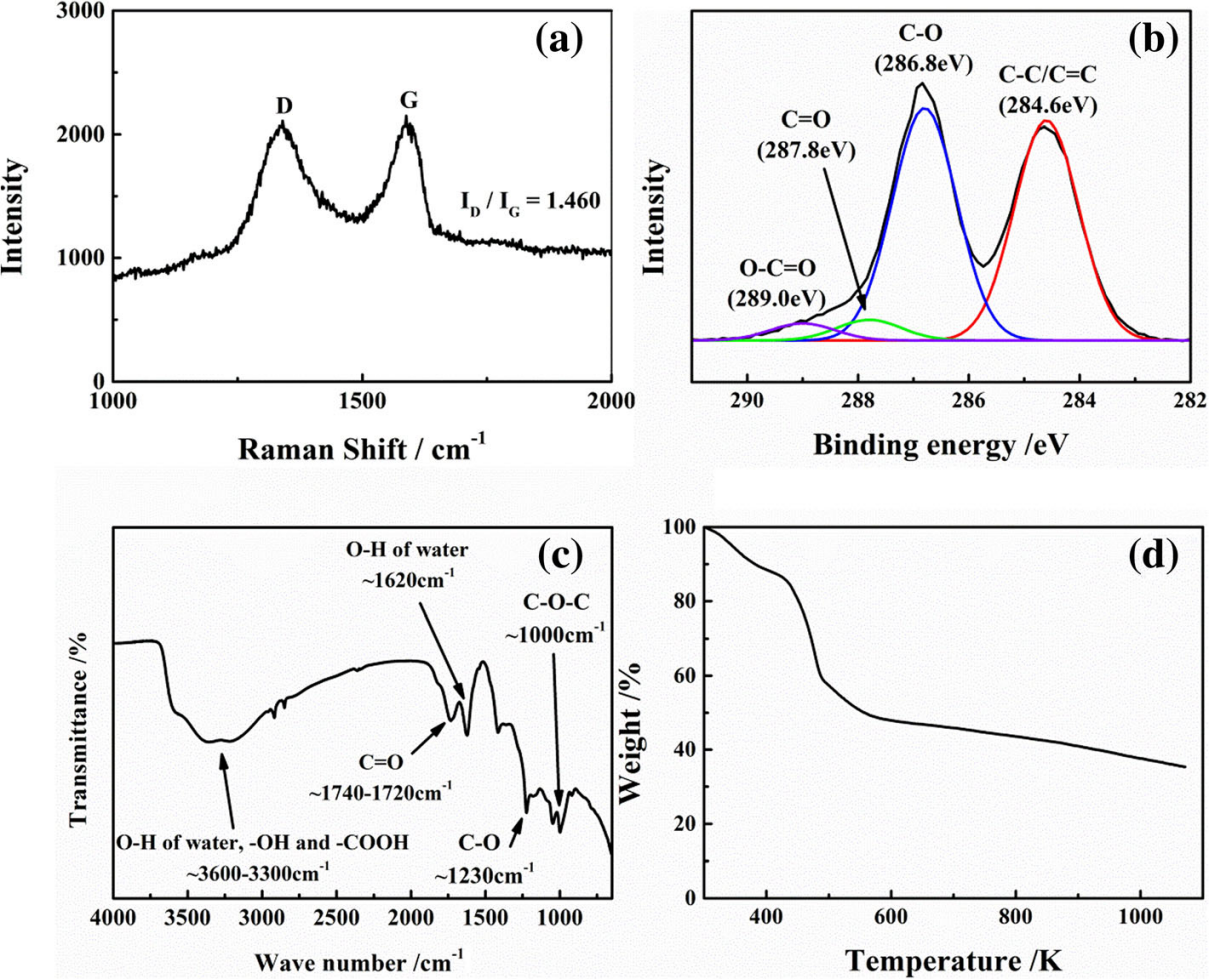
Structural characterization of as-prepared GO. **a** Raman spectra. **b** XPS C1s spectrum. **c** FT-IR spectra. **d** TGA plot

The structural characterization of the as-prepared GO is presented in Fig. 2. As shown in the Raman spectra in Fig. 2a, GO exhibits two distinct peaks at 1347 cm^−1^ (D-band) and 1582 cm^−1^ (G-band), which correspond to defects in the structure and degree of graphitization, respectively. The *I*_D_/*I*_G_ ratio represents the defect density for graphene material. In this study, the *I*_D_/*I*_G_ ratio is 1.460, indicating that many of the original sp^2^ bonds in graphite are replaced by the oxygen group induced during oxidation. To further reveal the chemical structure of GO, XPS analysis was performed, and the results are shown in Fig. 2b. The C1s XPS spectrum clearly indicates a considerable degree of oxidation of carbon atoms with different functional groups: the non-oxygenated ring C (C-C/C=C, ~284.6 eV), the C-O single bonds (C-O-H, C-O-C ~286.8 eV), the carbonyl (C=O, ~287.8 eV), and carboxyl (O-C=O, ~289.0 eV). The binding energy of different functional groups is marked in Fig. 2b. The aforementioned results are also revealed in FT-IR result, shown in Fig. 2c. A band at 3400 cm^−1^ is associated with the stretching vibration of the O-H bond. The peak at 1000 cm^−1^ corresponds to the stretching vibration of the C-O-C bond. In addition, the vibrations of C-O and C=O appear at the band with 1230 and 1730 cm^−1^ bandwidths, respectively. The TGA curves of GO are described in Fig. 2d. An apparent weight loss around 433 to 493 K is observed, which is ascribed to the decomposition of those unstable oxygen-containing functional groups. In addition, GO can be partly reduced during sintering.

**Fig. 3:**
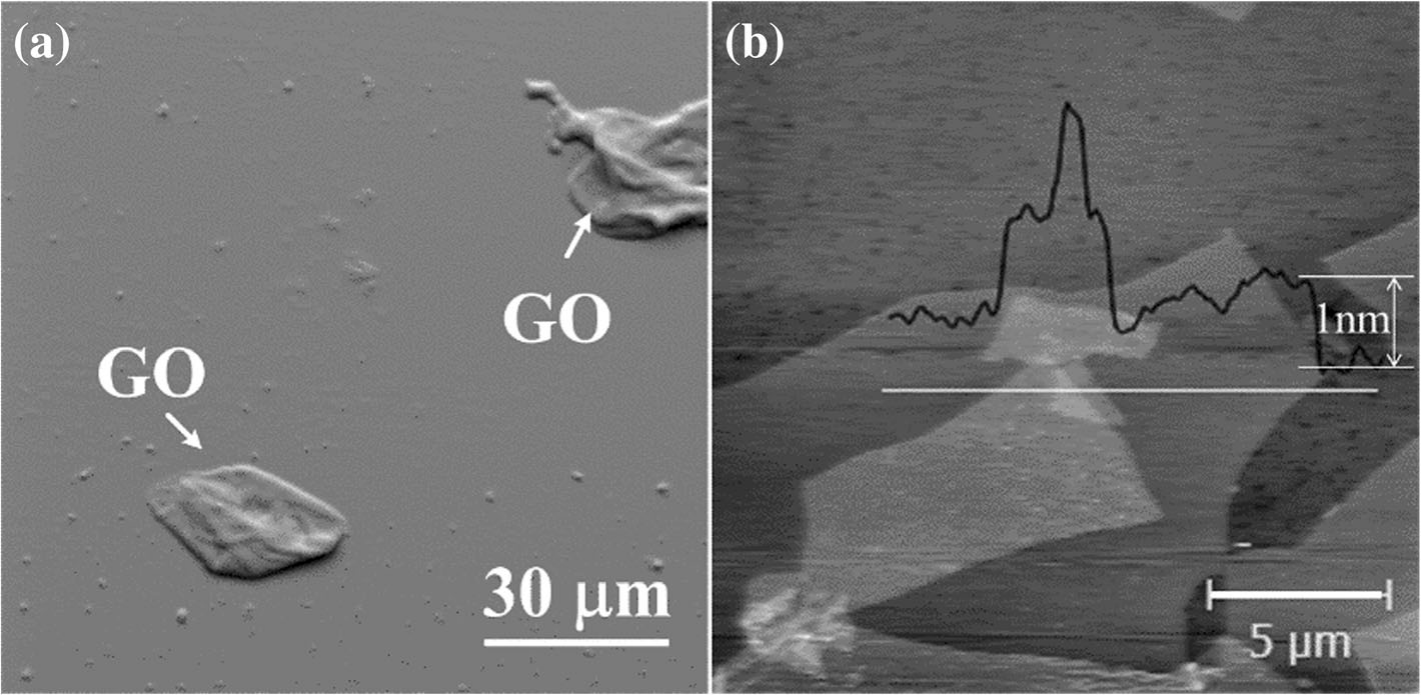
Microstructure of GO sheets prepared using the modified Hummers’ method. **a** SEM image. **b** AFM image

The microstructure of the GO sheets is presented in Fig. 3, which reveals a clear flat structure. The maximum size of the GO sheets is about dozens of micrometers, and the thickness is around 1 nm, as observed from the AFM image. These morphological results indicate that a single layer of polycyclic aromatic hydrocarbon structure has been successfully exfoliated from the graphite.

### Microstructural and Phase Analysis

**Fig. 4:**
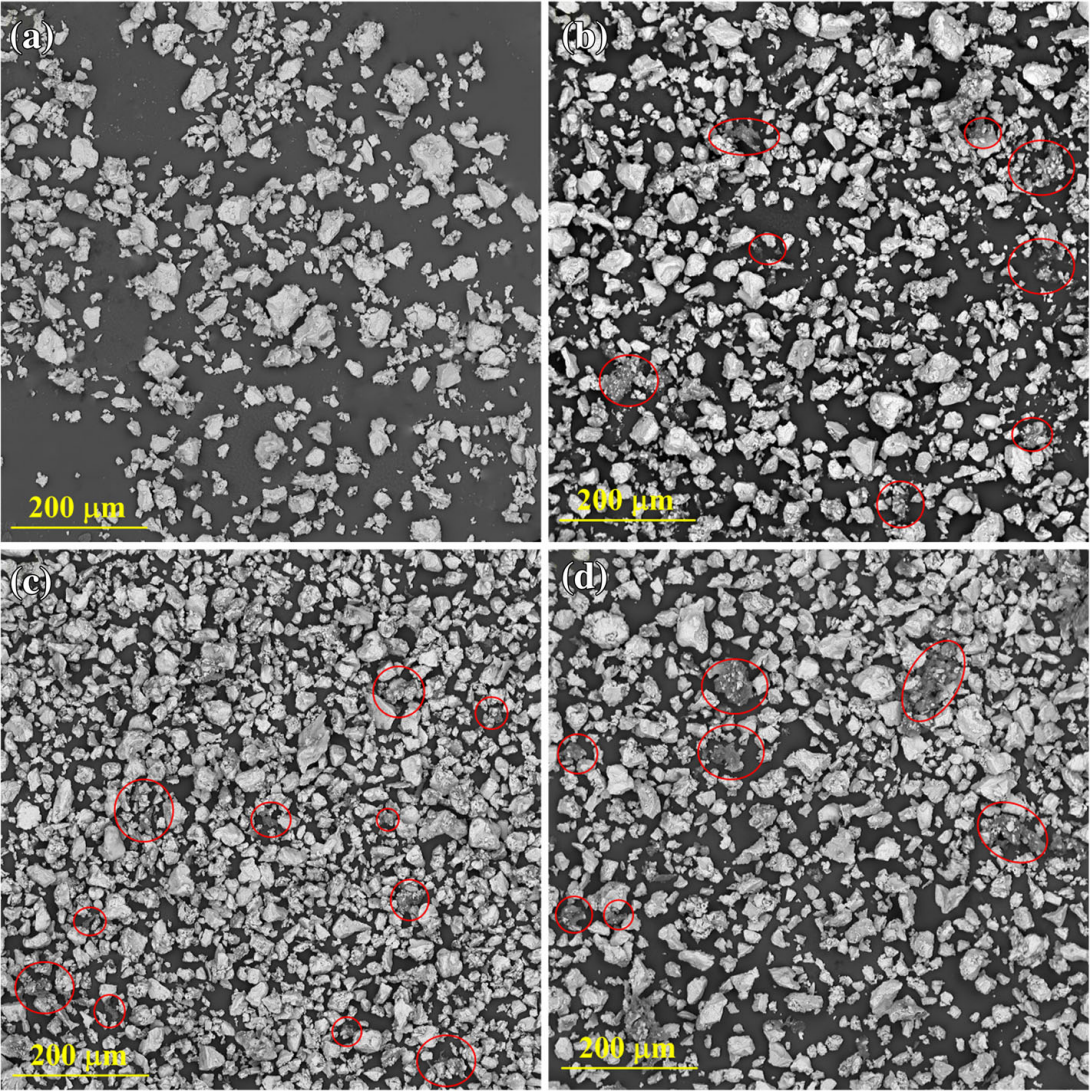
SEM micrographs of mixed powder of **a** pure Ti, **b** Ti-1 wt% GO, **c** Ti-2.5 wt% GO, and **d** Ti-5 wt% GO

The morphologies of the mixed composite powder with different GO contents are shown in Fig. 4. Small pieces of GO are marked by red circles. The GO is found to be uniformly distributed throughout the matrix. Most GO pieces are crimped and absorbed on the irregular surface of Ti powders. However, considerable aggregation also occurs when the GO content is increased to 5 wt%.

**Fig. 5:**
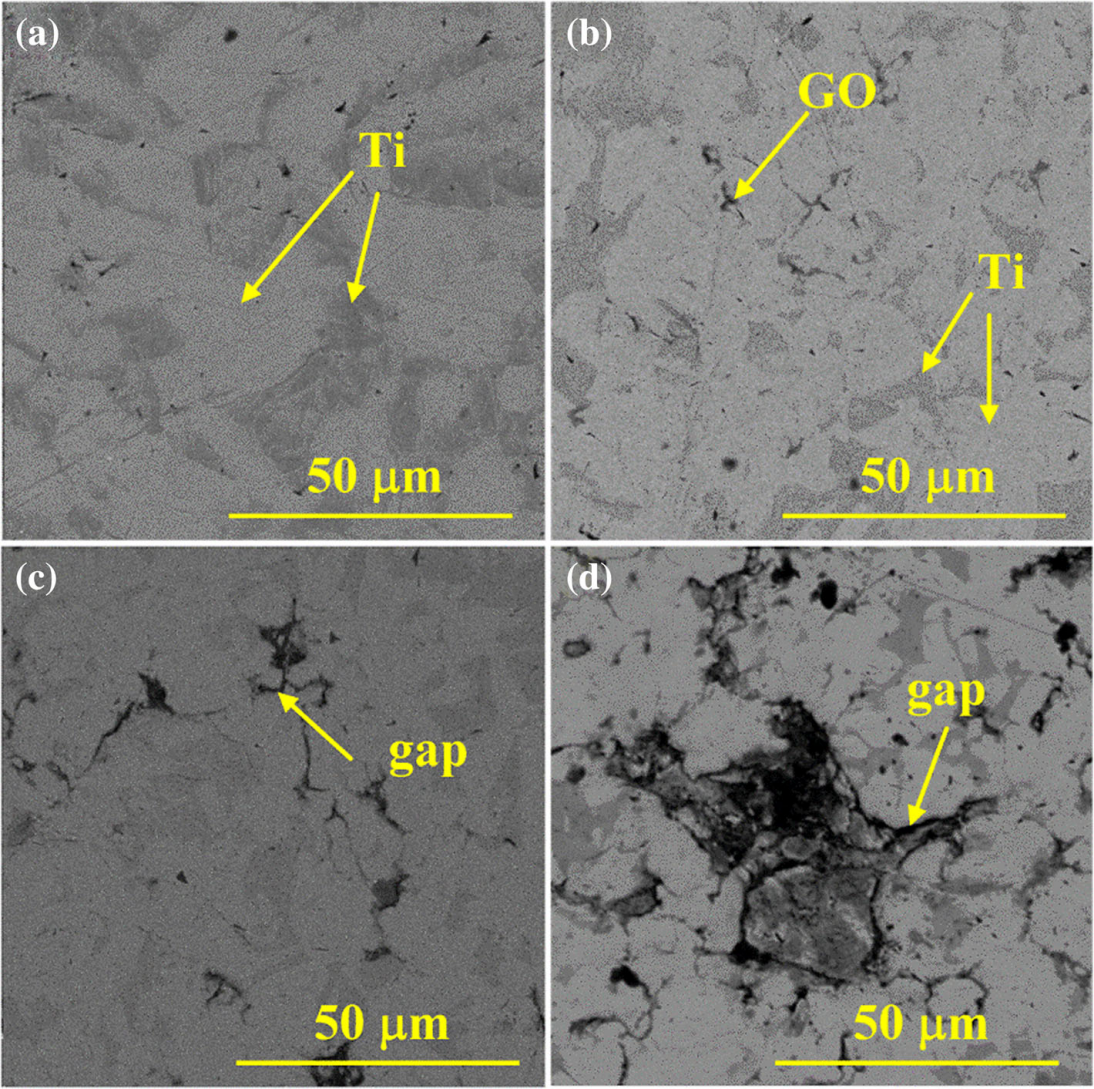
SEM micrograph of composites sintered at 1073 K with variations in GO contents. **a** Pure Ti. **b** Ti-1 wt%GO. **c** Ti-2.5 wt% GO. **d** Ti-5 wt% GO

**Fig. 6:**
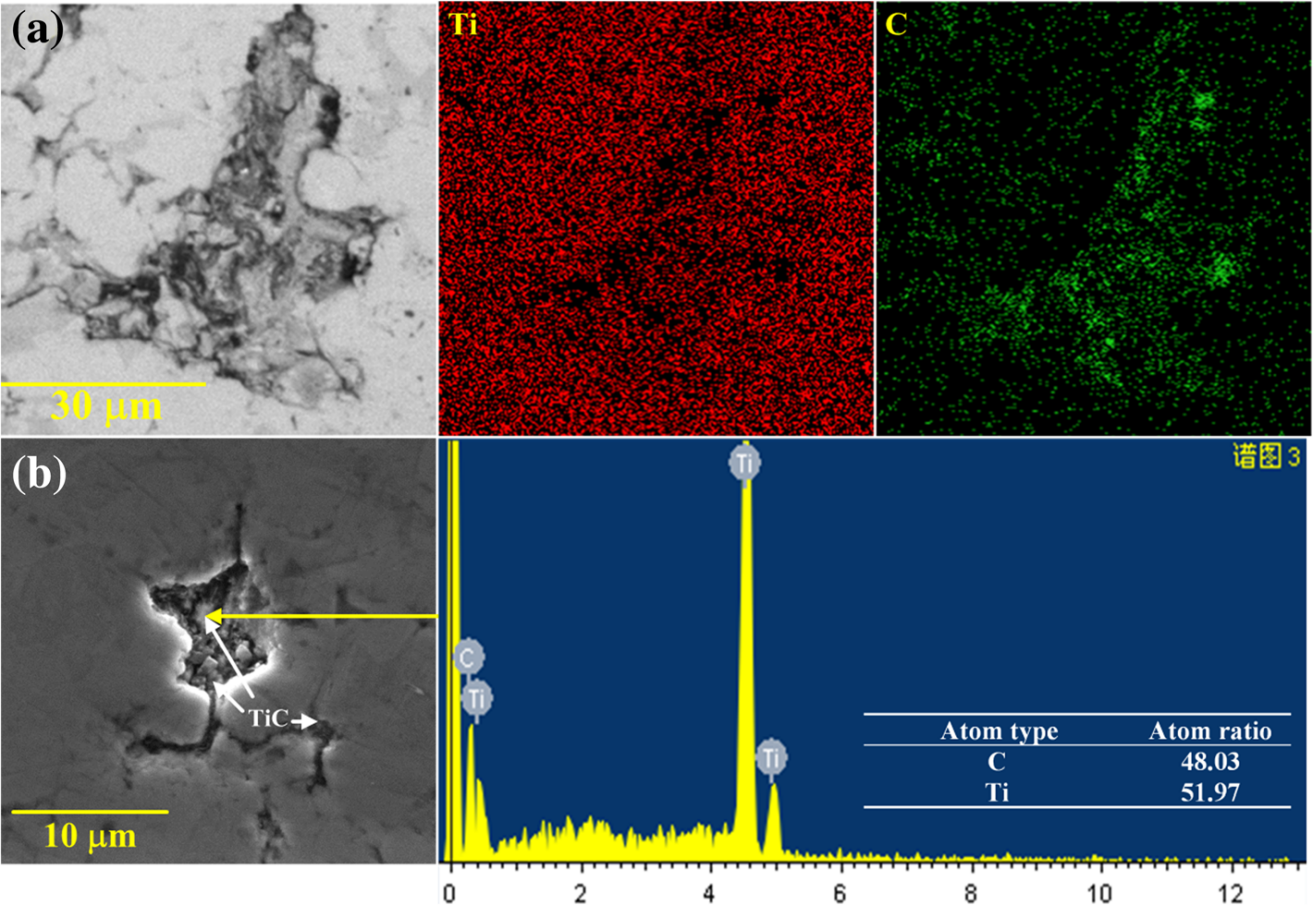
EDS analysis of composites sintered at 1073 K

Fig. 5 shows the surface micrographs of composites sintered at 1073 K with variation in GO content. The bond between the Ti particles improves the densification of the composites at high temperatures. The GO is uniformly distributed with a strip morphology in the Ti matrix. In addition, gaps and pores are markedly observed between the GO and Ti matrix because of low diffusion activation energy, which is caused by the decomposition of the oxygen-containing functional group of GO. The number of gaps increases with an increase in GO content. EDS results indicate that TiC particles are formed via the chemical reaction between the GO and Ti matrix during sintering and distributed at the gap edges, as shown in Fig. 6. The TiC formed in situ can not only improve the interfacial bonding between the Ti matrix and GO but also enhance the mechanical properties of the composite.

**Fig. 7:**
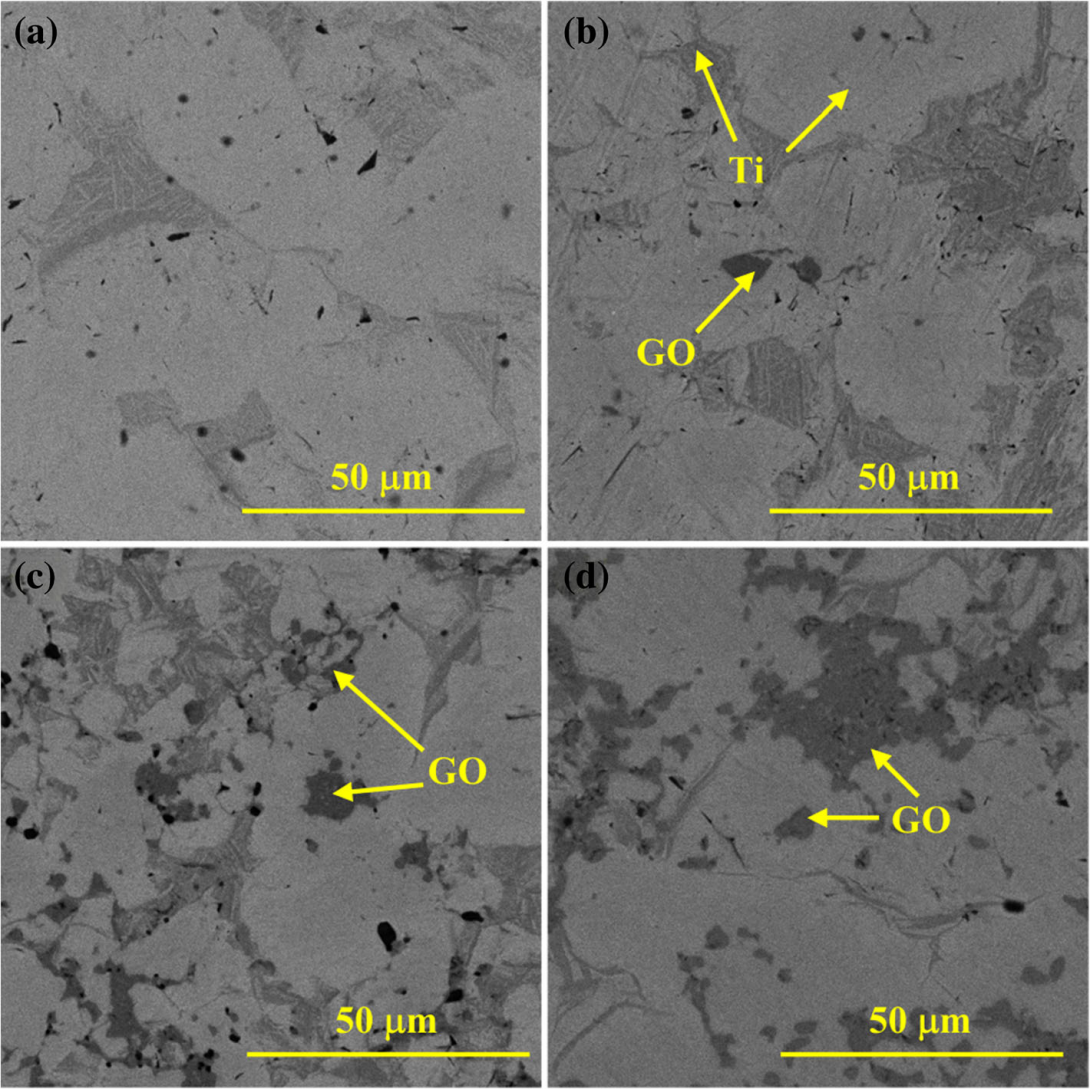
SEM micrograph of composites sintered at 1473 K with variation in GO content. **a** Pure Ti. **b** Ti-1 wt% GO. **c** Ti-2.5 wt% GO. **d** Ti-5 wt% GO

Morphologies of the Ti/GO composites sintered at 1473 K are shown in Fig. 7. Compared with the morphologies at 1073 K, the number of gaps markedly decreases, and the sample appears more compact as the temperature rises, which is attributed to the high diffusion efficiency of Ti. GO is also uniformly distributed in the matrix, and the amount of dispersoid increases with more GO added.

**Fig. 8:**
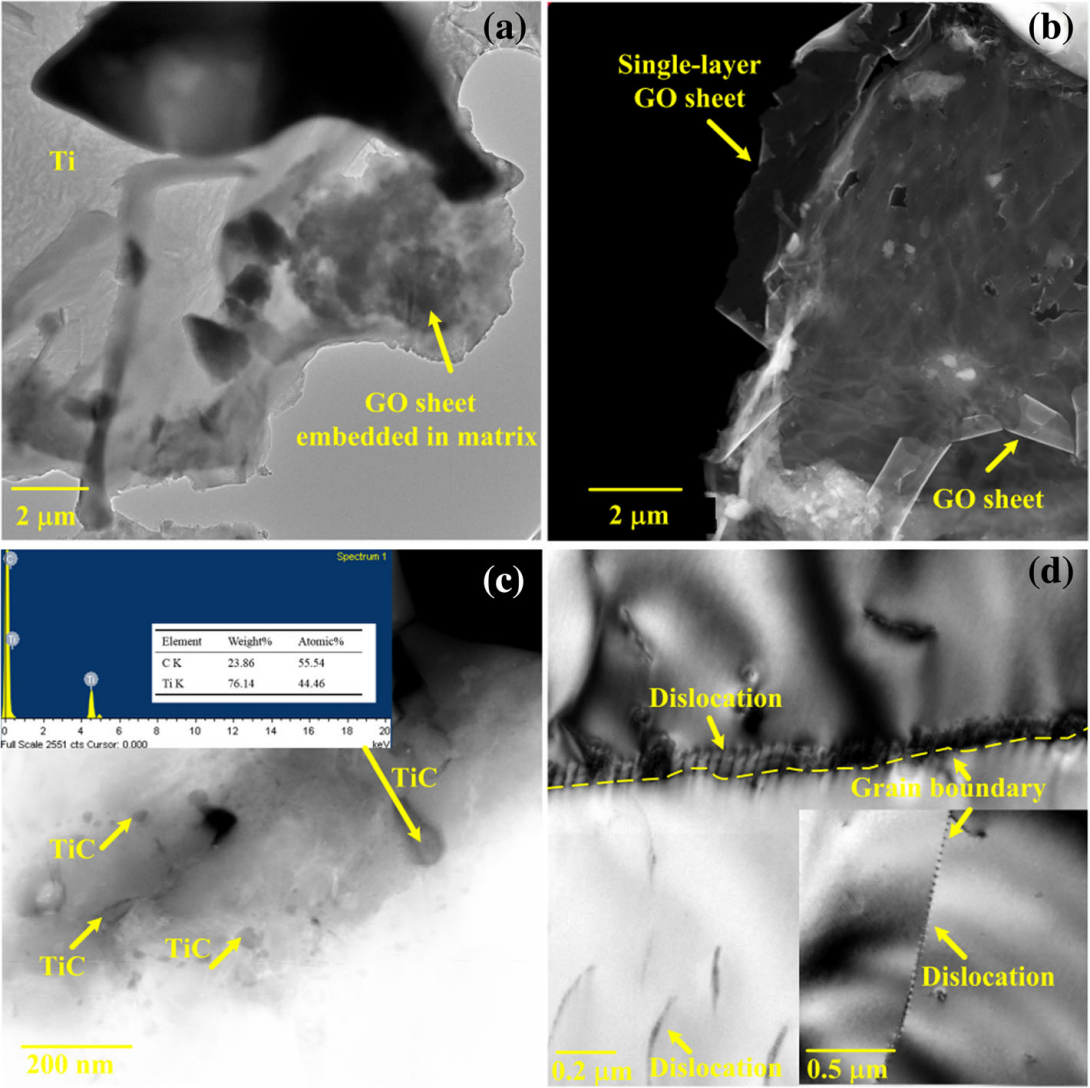
TEM micrographs of the Ti-2.5 wt% GO composite sintered at 1473 K. **a** Interface between GO sheets and Ti matrix. **b** Structure of GO retained in composites. **c** TiC formed in situ in composite. **d** Dislocation generation at the grain boundary

The TEM patterns of the Ti-2.5 wt% GO composite sintered at 1473 K are presented in Fig. 8. The bright-field image of the composite shows that the GO sheets embedded in Ti are attached in the boundaries of the Ti matrix, as shown in Fig. 8a. The single-layer flake structure of many GO sheets is partly retained by the dark-field TEM image, as shown in Fig. 8b. Some nano-size particles and pores appear on the GO sheets. Fig. 8c reveals the analysis of the microstructure and chemical components of TiC particles with sizes ranging from 20 to 200 nm, which are formed in situ between Ti and GO. The wide range of the TiC particle size is attributed to the variation in size of the GO sheets as the sole carbon source. This phenomenon is also reported in other studies. Zhang et al. [25] demonstrated that Ti/graphene composites form TiC particles with size ranging from 100 nm to 5 μm. Karthiselva and Bakshi [26] revealed that TiC rods with a diameter of 30 to 100 nm are formed in carbon nanotube-reinforced titanium diboride matrix composites. Gaps formed by the decomposition of inhomogeneous oxygen-containing functional groups in GO are also factors preventing further reaction between the Ti matrix and GO. In addition, numerous dislocations are generated in both grain boundaries and the grain, as shown in Fig. 8d. This occurrence is caused by the variation in the coefficient of thermal expansion (CTE) between the TiC particles, GO sheets, and Ti matrix. This difference leads to highly localized residual stress in the vicinity of nanofillers, creating dislocations. More dislocations are pinned in grain boundaries where GO sheets are gathered. High dislocation density results in dislocation tangles, increasing the strength of the composites.

**Fig. 9:**
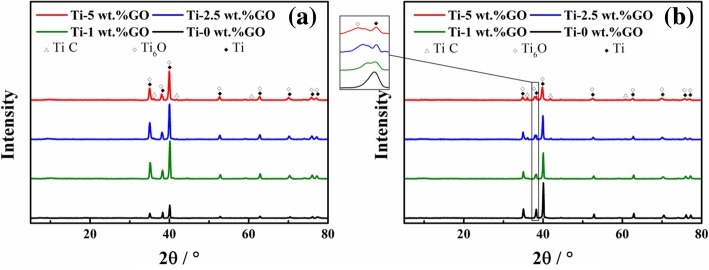
XRD patterns of composites sintered at **a** 1073 K and **b** 1473 K

XRD analysis of composites sintered at different temperatures is presented in Fig. 9. All samples have main Ti peaks at 2*θ* = 35.09° (1 0 0), 38.42° (0 0 2), and 40.17° (1 0 1). With the addition of GO, the weak diffraction peaks of titanium oxide and TiC gradually appear, indicating the occurrence of a chemical reaction between Ti and GO. The formation of titanium oxide is attributed to the oxygen-containing functional groups of GO. The standard free energy (Δ*G*) of TiC formation at 1073 K is − 178.87 KJ/mol, and that at 1473 K is − 177.26 KJ/mol, calculated based on the relationship between Δ*G* and *T* [27]. Thus, TiC is formed in situ by the reaction between Ti and C during sintering, which is consistent with the aforementioned SEM and TEM results. A similar occurrence was observed by Dong et al. [28]. The intensity of TiC increases with an increase in GO content. Notably, significant broadening of Ti peaks occurred and moves distinctly to the high 2*θ* angle with an increase in GO content, indicating the gradual decrease in grain size of the powder after the addition of GO.

### Mechanical Properties

**Fig. 10:**
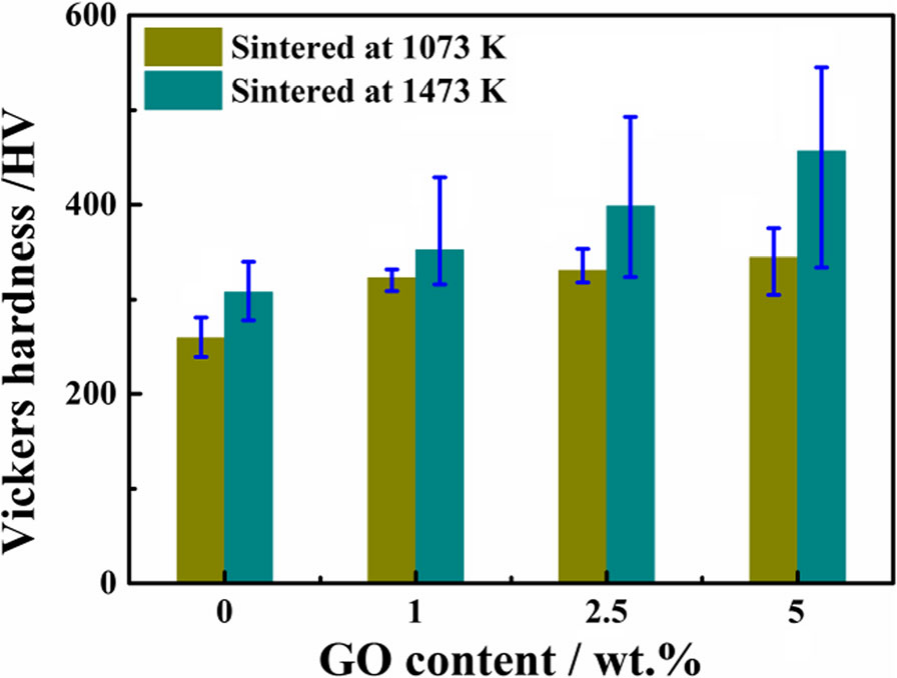
Vickers hardness of Ti/GO composites

The Vickers hardness of the Ti/GO composites was evaluated with a loading weight at 250 g and dwell time of 10 s. Measurements were performed at least five times for each sample with random surface locations, and the average value was determined. Fig. 10 shows the relationship between hardness and GO content at different sintering temperatures; hardness is enhanced with an increase in GO content. Compared with the pure Ti sample, the composite reinforced with 5 wt% GO has a hardness of 347 HV, which is 25.4% higher than that of the pure matrix at 1073 K. This result indicates that the addition of GO positively affects the hardness of the composites. With an increase in sintering temperature to 1473 K, hardness significantly increased relative to 1073 K for all composites, indicating that an elevated temperature can reduce the void and increase the density of the sample. Increased temperature also benefits the dynamic conditions of TiC formation, thereby increasing the hardness of the composite. As shown in Fig. 10, the hardness of the Ti-5 wt% GO composite increases from 344 to 457 HV when the sintering temperature increases from 1073 to 1473 K.

**Fig. 11:**
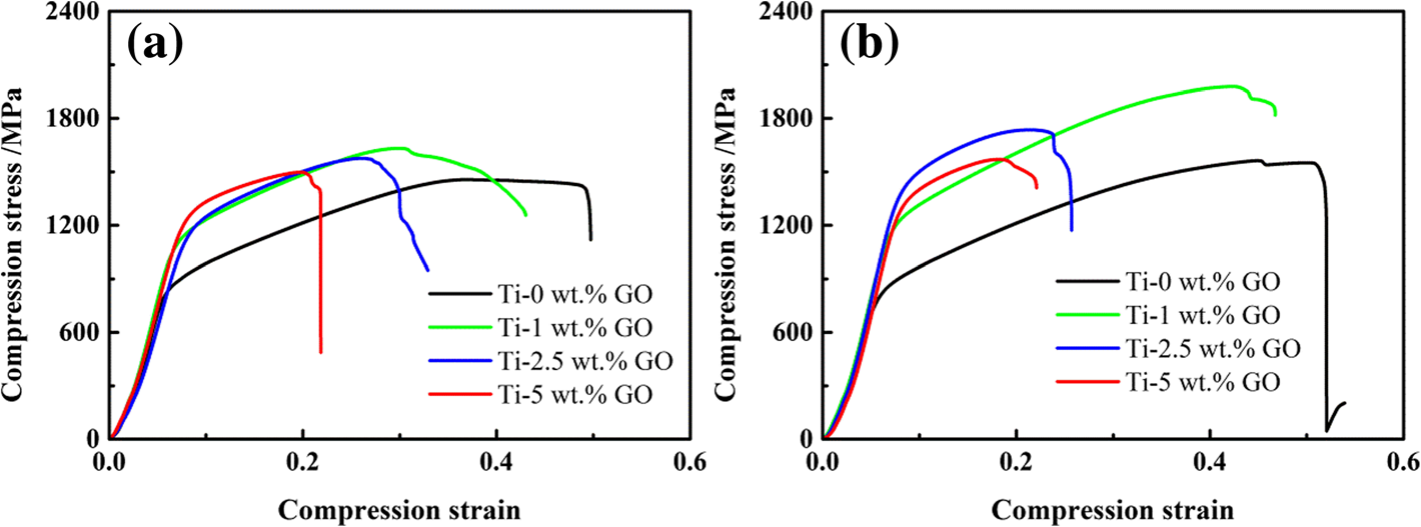
Stress-strain curve of samples sintered at **a** 1073 K and **b** 1473 K

The compressive stress-strain curves of the Ti/GO composite are plotted in Fig. 11 by compressing the cylindrical samples with a diameter of 4 mm and a height of 10 mm at a loading rate of 0.5 mm/min. The compression test results for pure Ti are used to compare and illustrate the significant increase in strength. As shown in Table 2, a substantial improvement in strength can be confirmed by the addition of GO. With the weight fraction of GO rising from 0 to 5 wt%, the yield stress of 1073 K sintered sample increases gradually. The Ti-5 wt% GO composite sintered at 1073 K exhibits a yield stress of 1173 MPa, which is 40.6% higher than that of pure Ti processed under identical conditions. Similarly, the yield stress of the samples sintered at 1473 K increased with an increase in the weight fraction of GO from 0 to 2.5 wt%. The Ti-2.5 wt% GO composite sintered at 1473 K exhibits yield stress equal to 1294 MPa, which is 62.7% higher than that of pure Ti. A further increase in GO content to 5 wt% leads to a slight decrease in yield stress owing to GO agglomeration, which is consistent with the result shown in Fig. 4(d). Similarly, the addition of GO leads to an increase in ultimate stress of both temperature-sintered samples. The ultimate stress of the Ti-1 wt% GO composite sintered at 1073 K and 1473 K are 1632 MPa and 977 MPa, which are 12% and 27% higher than that of pure Ti, respectively. In addition, the curves suggest that the temperature significantly influences the strength. The yield stress and ultimate stress of the composite with the same GO content are increased with an increase in sintering temperature. The ultimate stress and yield stress of Ti-2.5 wt% GO are 1736 and 1294 MPa at 1473 K, reflecting increment of 10.2% and 18.6% relative to that of the composite with the same GO content at 1073 K. As previously mentioned, an elevated temperature can promote sample densification and TiC formation, resulting in the enhanced strength.

**Table 2:** Mechanical properties of Ti/GO composites

Content of GO	1073 K		1473 K	
	Yield stress/MPa	Ultimate stress/MPa	Yield stress/MPa	Ultimate stress/MPa
0 wt%	834	1456	795	1561
1 wt%	1046	1632	1115	1977
2.5 wt%	1091	1576	1294	1736
5 wt%	1173	1498	1229	1569

### Fracture Analysis

**Fig. 12:**
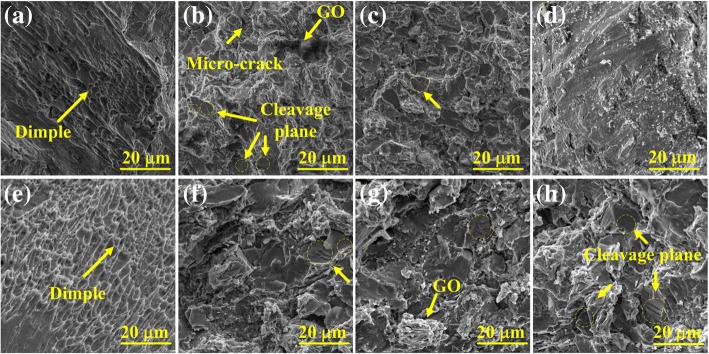
Fracture morphology of **a** pure Ti sintered at 1073 K, **b** Ti-1 wt% GO sintered at 1073 K, **c** Ti-2.5 wt% GO sintered at 1073 K, **d** Ti-5 wt% GO sintered at 1073 K, **e** pure Ti sintered at 1473 K, **f** Ti-1 wt % GO sintered at 1473 K, **g** Ti-2.5 wt% GO sintered at 1473 K, and **h** Ti-5 wt% GO sintered at 1473 K

The compression fracture morphologies of the samples sintered at different temperatures are shown in Fig. 12. Numerous dimples are evident for pure Ti samples at both sintering temperatures, as shown in Figs. 12a and e, exhibiting ductile fracture characteristics. The fracture analysis for the composite reinforced with GO reveals an obvious distinction of fracture characteristics compared with the Ti matrix. Several cleavage planes and microcracks appear in the Ti/GO composites, exhibiting quasi-cleavage fracture characteristics. In addition, these features become more apparent with an increase in sintering temperature. The size of the cleavage plane increases with rising sintering temperature because of the reduction in gaps and pores. These pores and gaps are responsible for the fracture and crack initiation.

### Thermal Analysis

**Fig. 13:**
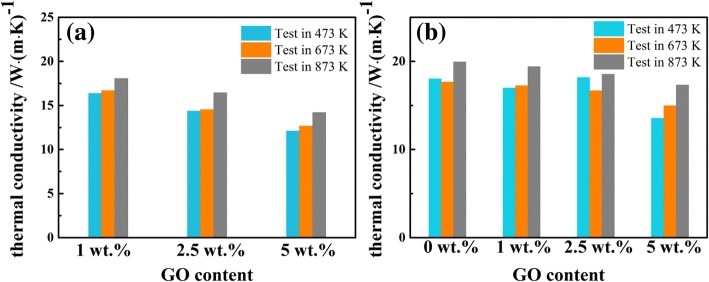
Thermal conductivity of composites sintered at **a** 1073 K and **b** 1473 K

The thermal conductivity of the Ti/GO composite was evaluated at 473 K, 673 K, and 873 K by using a small round planchet measurement sample with 8 mm in diameter and 1 mm in height, and the results are shown in Fig. 13. The thermal conductivity decreases with an increase in GO content, indicating that the addition of GO can deteriorate the thermal conductivity of the composite. This result is attributed to the poor thermal conductivity of GO and the incomplete reduction in GO. In addition, the thermal conductivity is prevented by the gaps between the matrix and GO resulting from the decomposition of oxygen-containing functional groups. Therefore, the thermal conductivity of the composite cannot be improved by the addition of GO. Figure 13 also reveals that the thermal conductivity of the Ti/GO composite markedly increases with an enhancement in sintering temperature. The reason is that the gaps are reduced and the sample compactness is increased as the sintering temperature rises. A greater amount of GO is reduced to graphene at a high temperature, resulting in a higher thermal conductivity than that sintered at 1073 K. The thermal conductivity of the Ti/GO composites may also be deduced to improve with the addition of GO if GO is chemically reduced to graphene first before sintering.

### Strengthening Mechanism

Grain refinement, solution strengthening, and dispersion strengthening of TiC and GO are considered as the main strengthening mechanism in this study in accordance with the aforementioned results. With an increase in GO content, the grain size is refined. The refinement of grain size represents more intensive grain boundaries, which prevent dislocation motion and induce an increase in the yield strength of materials. This contribution to strength is described by the widely known Hall-Petch relationship [29, 30], as follows:

5 $$ \sigma ={\sigma}_0+k{D}^{-\frac{1}{2}} $$

where *σ* and *σ*_0_ are the yield stress and friction stress when dislocations glide on the slip plane, respectively. *k* is the stress concentration factor, which is related to the material only. *D* is the average grain size. The value of *k* is associated with the number of the slip system. This value is higher for the hexagonal close packing (HCP) metals than face-centered cubic (FCC) and body-centered cubic (BCC) metals [31]. Ti exhibits an HCP structure; thus, grain refinement significantly increases yield strength.

Solid solution strengthening is also considered a crucial strengthening mechanism. Owing to their substantial difference in atom radius, carbon and oxygen are effective solute atoms for the Ti matrix. The solute atoms can cause lattice distortion and pin dislocation motion to improve the yield stress of the material.

The GO and TiC formed in situ are uniformly dispersed in the Ti matrix. These dispersive TiC nanoparticles can effectively strengthen composites. The high dislocation density is generated by different thermal expansivity between the Ti matrix and the reinforcements. Orowan strengthening [32] is also regarded as an important strengthening mechanism; dislocation motion consumes far more energy to bypass GO sheets with high specific surface area.

## Conclusions

GO with varying content—1 wt%, 2.5 wt%, and 5 wt%—were employed as reinforcements to prepare titanium matrix composites by hot-pressed sintering at different temperatures in this study. The following conclusions are drawn:

1. GO is uniformly distributed in the matrix when the content is lower than 5 wt%. TiC measuring 20–200 nm is formed in situ as an interfacial product by the reaction between Ti and GO during sintering. With increases in GO content and sintering temperature, the amount of TiC nanoparticle in situ increases. In addition, GO is partly retained, with a lamellar structure after sintering.
2. Hardness, yield strength, and ultimate strength are significantly improved by the addition of GO and sintering temperature. The Ti-5 wt% GO composite has a maximum hardness of 457 HV, which is 48.4% higher than that of pure Ti at 1473 K. The Ti-2.5 wt% GO composite sintered at 1473 K shows a peak yield stress of 1294 MPa, which is 62.7% higher than that of pure Ti because of GO agglomeration in the Ti-5 wt% GO composite.
3. The Ti/GO composites exhibit a quasi-cleavage fracture instead of a ductile fracture for the pure Ti matrix. With a rising sintering temperature, the size of the cleavage plane increases. The thermal conductivity of the composite is deteriorated by the addition of GO but improved with an increase in sintering temperature.
4. The grain refinement, solution strengthening, and dispersion strengthening of GO and TiC in situ are the main strengthening mechanisms of the Ti/GO composites in this study.

## Abbreviations

AFM: Atomic force microscopy
BCC: Body-centered cubic
CEG: Chemically expanded graphite
CTE: Coefficient of thermal expansion
EDS: Energy-dispersive spectrometer
FCC: Face-centered cubic
FT-IR: Fourier-transform infrared spectroscopy
GIC: Graphite intercalation compound
GNFs: Graphene nanoflakes
GO: Graphene oxide
HCP: Hexagonal close packing
MMCs: Metal matrix composites
SEM: Scanning electron microscope
TEM: Transmission electron microscopy
TGA: Thermogravimetric analysis
TMCs: Titanium matrix composites
XPS: X-ray photoelectron spectroscopy
XRD: X-ray diffraction
